# Changes in Body Reserves, Non-Esterified Fatty Acids, and Leptin during the Reproductive Lifespan of the Rabbit Female

**DOI:** 10.3390/ani13203213

**Published:** 2023-10-14

**Authors:** Rosa Peiró, María-José Argente, María-Luz García

**Affiliations:** 1 Instituto de Conservación y Mejora de la Agrodiversidad Valenciana (COMAV), Universitat Politècnica de València, Camino de Vera s/n, 46022 Valencia, Spain; 2 Centro de Investigación e Innovación Agroalimentaria y Agroambiental (CIAGRO-UMH), Universidad Miguel Hernández de Elche, Ctra. Beniel km 3.2, 03312 Alicante, Spain; mj.argente@umh.es (M.-J.A.); mariluz.garcia@umh.es (M.-L.G.)

**Keywords:** body weight, delivery, leptin, perirenal fat thickness, mating, NEFA

## Abstract

**Simple Summary:**

The productivity and survival of females in the rabbit industry depends largely on the correct management of their body reserves. This management includes both their body condition and the mobilization of their body reserves during high energy demand or critical moments for reproduction, such as mating, gestation, or delivery. In this work, a study of body weight, perirenal fat thickness, non-esterified fatty acid concentration, and leptin concentration at these key moments, when a semi-intensive reproductive rhythm is applied, is carried out. This detailed knowledge is decisive to optimising the productivity of females.

**Abstract:**

The aim of this work is to study changes in body weight, perirenal fat thickness (PFT), and non-esterified fatty acid (NEFA) and leptin concentrations throughout the reproductive life of the rabbit female and their correlations when a semi-intensive reproductive rhythm is applied. A total of 46 lactating females were used. Body weight, PFT, and NEFA and leptin concentration were recorded at 12 weeks of age, at first mating and delivery, and at second, third, and fourth mating, 12th d of gestation, and delivery. The highest body weight was detected on the 12th d of any gestation, around 4280 g, and the lowest weight was at delivery, around 4030 g. PFT increased until third mating. NEFA and leptin concentration showed a cyclical pattern throughout the reproductive lifespan of the females. NEFAs presented the highest concentration at delivery within each reproductive cycle and levels decreased over the course of the deliveries (0.423 mmol/L at first delivery, 0.406 mmol/L at second delivery, 0.371 mmol/L at third delivery, and 0.309 mmol/L at fourth delivery). Similar NEFA concentrations at mating and on the 12th d of gestation were obtained. Leptin showed the highest concentrations at mating within each reproductive cycle. Leptin decreased between mating and delivery in all reproductive cycles and it was close to 1 ng/mL HE. Low or null correlations were shown between body weight, PFT, and NEFA and leptin concentration at mating, 12th d of gestation, and delivery. In conclusion, females are able to maintain a semi-intensive reproductive rhythm across four parities weighing around 4 kg from first mating. Females had an increased perirenal fat thickness until third delivery, and their NEFA concentration was maximum at delivery and leptin concentration was maximum at mating. Body weight, PFT, and NEFA and leptin concentration should be measured during critical moments of reproductive life in order to determine body condition and energy mobilization, due to their low or null correlations.

## 1. Introduction

The determination of body condition and the measurement of energy mobilization in rabbit females is essential for research and technical and commercial purposes, to optimise their productivity. While body weight and perirenal fat thickness (PFT) are good predictors of body condition, NEFA measurements should be used when an accurate measurement of energy mobilization is needed [1].

Nowadays, several methods to evaluate the body condition in vivo during lifespan are available for rabbits. Also, a non-invasive method would be desired for animal welfare protection purposes. Measurement of the thickness of the main reserve tissue in the rabbit (perirenal fat) at a fixed anatomical location (8th–9th thoracic vertebrae [2]; 2nd and 3rd lumbar vertebrae [3]) using ultrasound scanning is a simple, low-cost, and accurate method [1]. The total body electrical conductivity method combines a measurement of body conductivity with the animals’ weight to estimate their composition [4]. The body condition score has also been evaluated using manual palpation [5,6]. A more recent method has been developed by Saiz et al. [7], and it consists of bioelectrical impedance analysis. Body condition estimation with the application of these techniques evaluated the effects of feeding programs on nulliparous females [8,9] and primiparous females [10,11], the influence of oocyte quality and follicular characteristics in primiparous females [12], effects on receptivity and fertility [13], the relationships between the reproductive traits in multiparous females [14], and the effect of the genetic line [13,15]. Moreover, it is well known that number of foetuses and the number of lactating kits can affect body condition during gestation and lactation [15,16,17].

Body reserve mobilization is reflected in changes in some metabolic parameters, such as non-esterified fatty acid (NEFA) and leptin concentrations [18]. NEFAs act at the ovarian level by modifying endocrine, paracrine, and autocrine regulation, which permits follicle growth, ovulation, and the development of the corpus luteum [19]. Leptin is a cytokine encoded by obese genes and preliminarily secreted by adipocytes [20], involving the control of satiety and energy metabolism. Leptin may act as the critical link between adipose tissue and the reproductive system, indicating whether adequate energy reserves are present for normal reproductive function [21]. Studies to date on NEFAs and leptin are related to feeding programs [22,23,24], metabolic adaptation to fasting [11,25], and oocyte quality and follicular characteristics [12]. Recently, leptin concentration has been related to the reproductive function in rabbit females in terms of ovulation rate and fetal development [26].

The commercial use of artificial insemination in rabbit farming is relatively recent, and it has motivated the introduction of new reproductive rhythms in rabbit production [27,28]. Comparative studies of the productivity of different reproductive rhythms have shown that the reproductive lifespan of the females is negatively affected and litter viability decreases when using intensive reproductive management (mating 4 d post-partum) compared to semi-intensive and semi-extensive management (mating 11 d and 18 d post-partum, respectively) [29,30]. On the contrary, an extensive rhythm (mating after weaning) provides a low number of parities per year, and low productivity causes excessive fatness in the females and thus an impairment of their reproductive performance [31]. Thus, 11 d post-partum mating corresponds to one of the most used reproductive rhythms in current rabbit production [32]. However, to date, it has not been monitored how the rabbit females manage their body reserves and energy mobilisation throughout their productive life to ensure their survival, as well as their productivity. An understanding of the variation in body condition and energy mobilization could improve the productivity of rabbit females under this reproductive rhythm.

Therefore, the objective of this study was to understand changes in body weight, perirenal fat thickness, and NEFA and leptin concentration and their correlation throughout the reproductive life of the rabbit female during four reproductive cycles when a semi-intensive reproductive rhythm was applied.

## 2. Materials and Methods

### 2.1. Animals

The rabbit females belonged to a cross-population of two lines divergently selected by uterine capacity [33]. Both lines were derived from the V line and selected by litter size at weaning [34]. The females were held on the experimental farm at the University Miguel Hernández de Elche (Spain). The building has a side-swept forced ventilation system. The air enters from the side of the building through 4 wet cooling panels (0.85 m × 1.20 m). On the opposite side of the panels, there are 4 fans that extract the air from each of the halls; these fans are automatically regulated depending on the ambient temperature. All animals were reared in individual ergonomic multi-purpose cages made of galvanised steel. The animals were housed in 34 cm wide, 43 cm long, and 31 cm high cages from 9 weeks of age until 17 weeks and in 33 cm wide, 90 cm long, and 37.5 cm high cages during the productive stage. Females were fed ad libitum two different commercial diets throughout the experiment. The first diet was provided from 9 weeks until the first mating (163 g crude protein, 212 g crude fibre, and 25 g ether extract per kg of feed; CUNICEBIAL, NANTA S.A., Las Palas, Murcia, Spain). From the first mating to the end of the trial, the second diet for reproductive rabbit females was provided (169 g crude protein, 157 g crude fiber, and 30 g ether extract per kg of feed; CUNILACTAL, NANTA S.A., Las Palas, Murcia, Spain). The diet was supplemented with coccidiostats. Animals had free access to water. During the study, no sanitary treatment was applied. The photoperiod was 16 h light: 8 h dark.

Females were mated first at 17 weeks of age and thereafter at 11 d after each delivery, so a semi-intensive reproductive rhythm was applied [32]. Females were brought into the male’s cage and mating was verified to have taken place. Mating was carried out weekly. A diagnosis of pregnancy was made using abdominal palpation 12 days after mating and non-pregnant females were taken back to mate 14 days after the previous mating. 

A total of 80 females started the experiment, but 34 of them were removed from the experiment due to death at delivery (10 females), three non-receptive matings (12 females), and two failed palpations (12 females). Thus, 46 females were controlled until the fourth reproductive cycle and all were lactating at mating.

The litter size at birth was recorded. The mean and the standard deviation were 9.08 (2.95) at first parity, 8.83 (3.00) at second parity, 9.23 (2.84) at third parity, and 9.35 (2.81) at fourth parity. 

At delivery, the litters were equalised at nine kits. Kits were weaned at 28 d of age. The females were allowed to enter the nest box and suckle their litters once daily (09:00 h) for a maximum of 20 min.

### 2.2. Experimental Design

#### 2.2.1. Body Condition

Body weight and perirenal fat thickness (PFT) were measured at 12 weeks of age, at 1st mating and delivery, and at 2nd, 3rd, and 4th mating, the 12th day of these gestations, and their deliveries.

The hair was removed around the 8th and 9th thoracic vertebrae by shearing it, and ultrasound gel was applied to the scanning sites. Females were situated in a box during ultrasound measurement. Two measurements were made on the right sides of the back 3 cm from the vertebral column [2].

Perirenal fat thickness was measured using ultrasound imaging as described [26], using JustVision 200SSA-320A Toshiba ultrasound equipment.

#### 2.2.2. Serum Parameters

Blood samples were collected from the margin ear vein into tubes containing EDTA (ethylenediaminetetraacetic acid) at 1st mating and delivery and at 2nd, 3rd and 4th mating, 12th day of these gestations, and their deliveries. Immediately, plasma was obtained after centrifugation at 3000× *g* for 15 min at 4 °C and stored at −20 °C until the assays for NEFA and leptin were performed.

Duplicate plasma aliquots for the sample tubes were assayed. The non-esterified fatty acid (NEFA, mmol/L) concentrations were analyzed in duplicate using two reactions in enzymatic-based colorimetric assay from WAKO (NEFA-C ^®^, Wako Chemicals GmbH, Neuss, Germany) based on the ability of NEFA to acylate coenzyme A in the presence of CoA synthetase. The leptin concentrations were measured using the antibody RIA with a multi-species leptin kit (XL-85K, Linco Research Inc.^®^, St. Charles, MI, USA) as previously reported by García et al. [20]. The intra-assay variabilities were in all instances below 3% (CV); inter-assay variations were in all instances below 5.5% (CV).

### 2.3. Statistical Analysis

Body weight was analysed with the following model:

y_ijk_ = RS_i_ + F_ij_ + e_ijk_, (1)

where RS_i_ was the fixed effect of the reproductive status with 12 levels (12 weeks of age, at 1st mating and delivery, and at 2nd, 3rd and 4th mating, the 12 d of these gestations, and their deliveries); F_ij_ was the random effect of the females and e_ijkl_ was the error. 

PFT was analysed with the following model:

y_ijk_ = b × BW + RS_i_ + F_ij_ + e_ijk_, (2)

where b × BW was body weight as the covariate.

The model applied to analysing the plasma NEFA and leptin concentrations was the following:

y_ijk_ = RS_i_ + F_ij_ + e_ijk_, (3)

where RS_i_ was the fixed effect of the reproductive status with 11 levels (at 1st mating and delivery and at 2nd, 3rd and 4th mating, the 12 d of these gestations, and their deliveries); F_ij_ was the random effect of the females and e_ijkl_ was the error. 

Correlations between the residuals of a model that included the female effect, and mating, gestation, or parity order effect were estimated for data at mating, the 12th day of gestation, and parity, respectively.

The traits were analyzed using variance analysis using a mixed procedure (PROC MIXED), and residual correlations were estimated (PROC CORR) using the statistical package SAS (SAS Institute, 2022, Cary, CA, USA).

## 3. Results

**Figure 1:**
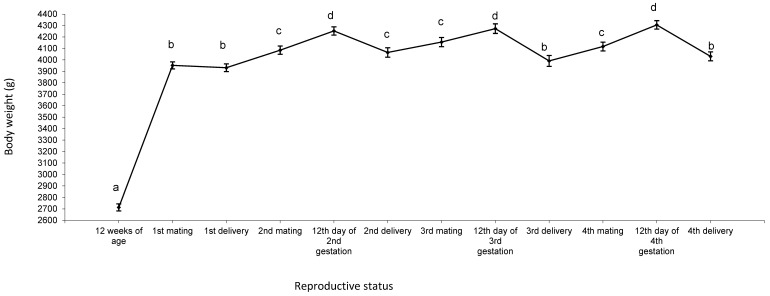
Changes in body weight of 46 females during four reproductive cycles. ^a,b,c,d^ Different letters indicate differences between means (*p* < 0.05).

The changes in body weight from 12 weeks to fourth delivery, when a semi-intensive reproductive rhythm was applied (i.e., mating took place 11 d after delivery), are presented in Figure 1. Body weight rose sharply from 12 weeks of age (2700 g) until the first mating (3905 g). Body weight increased between the first delivery and the subsequent mating, by around 150 g, and between the second mating and the 12th d of gestation of this gestation, by around 170 g. After, the highest body weight was detected on the 12th d of any gestation (4280 g), and the lowest weight was observed at delivery (4030 g). Although females varied in weight through their lifespans, they weighed around 4000 g at first mating and at fourth delivery.

**Figure 2:**
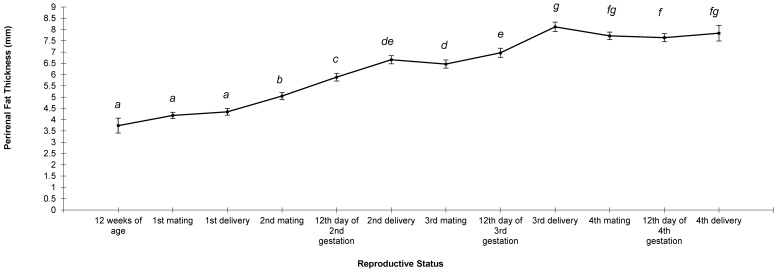
Changes in perirenal fat thickness of 46 females during four reproductive cycles. ^a,b,c,d,e,f,g^ Different letters indicate differences between means (*p* < 0.05).

PFT was 3.74 mm at 12 weeks of age for the females, 4.19 mm at the first mating, and 4.35 mm at the first delivery (Figure 2). By the second reproductive cycle, females had an increased PFT (0.70 mm between first delivery and mating, 0.84 mm between mating and 12th d of gestation, and 0.77 mm between 12th d of gestation and second delivery). The PFT was similar between the second delivery and third mating, but the PFT increased on the 12th d of the third gestation (6.97 mm) and at third delivery (8.12 mm). By the fourth reproductive cycle, the females did not change in their PFT (around 7.70 mm).

**Figure 3:**
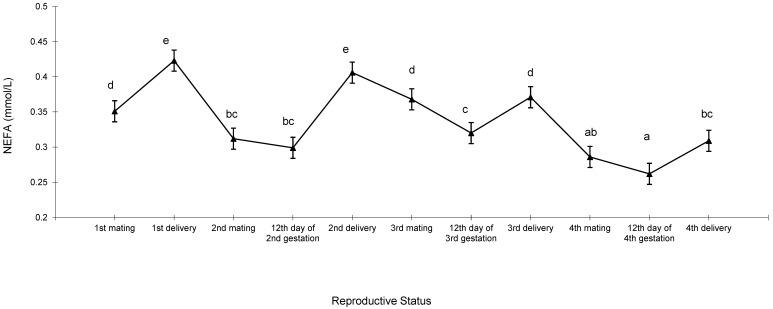
Changes in non-esterified fatty acids (NEFAs) of 46 females during four reproductive cycles. ^a,b,c,d,e^ Different letters indicate differences between means (*p* < 0.05).

NEFA concentration followed a cyclical pattern throughout the reproductive lifespan of the females (Figure 3). NEFA presented the highest concentration at delivery within each reproductive cycle and its levels decreased over the course of the deliveries. (0.423 mmol/L at first delivery, 0.406 mmol/L at second delivery, 0.371 mmol/L at third delivery, and 0.309 mmol/L at fourth delivery). The NEFA concentrations ranged between 0.368 mmol/L and 0.262 mmol/L, when females were mated or on the 12th d of gestation.

**Figure 4:**
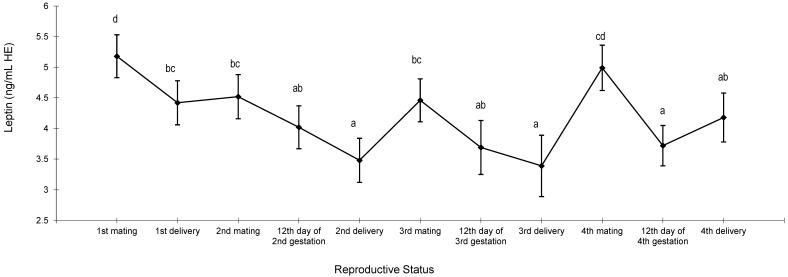
Changes in leptin of 46 females during four reproductive cycles. ^a,b,c,d^ Different letters indicate differences between means (*p* < 0.05).

Leptin also showed a cyclical pattern, but the highest concentrations were at mating within each reproductive cycle (Figure 4). The leptin concentrations decreased between mating and delivery in all reproductive cycles and it was close to 1 ng/mL HE (5.18 ng/mL HE vs. 4.42 ng/mL HE at first reproductive cycle; 4.52 ng/mL HE vs. 3.48 ng/mL HE at second reproductive cycle; 4.46 ng/mL HE vs. 3.39 ng/mL HE at third reproductive cycle; 4.99 ng/mL HE vs. 4.18 ng/mL HE fourth reproductive cycle, Figure 4). There was no difference between the first delivery and second mating, whereas an increase was observed in the last reproductive cycles. A similar concentration was found on the 2nd d of gestation and delivery in the valuated reproductive cycles.

The correlations between PFT, body weight, NEFA and leptin concentrations at mating and on the 12th d of gestation, and parity are displayed in Table 1 and Table 2. A positive and low correlation was found between PFT and body weight at all reproductive stages and it ranged from 0.222 to 0.249. The correlation between leptin and body weight was also positive; 0.342 at mating, 0.220 on the 12th d of gestation, and 0.264 at parity. Leptin and PFT were only positively correlated at mating (0.238). NEFA concentration was not correlated with PFT, body weight, or leptin concentration at any reproductive stage.

## 4. Discussion

The body condition and energy balance of females in commercial rabbit farms are generally critical. A semi-intensive reproductive rhythm involves mating being realised 11 d post-partum. If weaning is conducted 28 d post-partum, a high overlap between the beginning of gestation and the last part of lactation is produced. Thus, females overlap 61% of lactation and 57% of gestation. Females that maintain this reproductive rhythm, characterised by a high lactation–gestation overlap, should be able to manage properly their body reserves and energy resources.

Sixty per cent of females in our study were able to maintain this reproductive rhythm across four parities. They were characterised by the following profile of body reserves and energy mobilisation: maintaining a weight close to 4 kg, increased perirenal fat thickness until the third delivery, and maximised NEFA and leptin concentrations at delivery and at mating, respectively.

An adult body weight is achieved by the second reproductive cycle (4100 g), which is equivalent to 30 weeks of age, as reported in females of the maternal line by Blasco and Gómez [35]. Moreover, Cardinali et al. [14] determined the optimal body condition in maternal lines was reached in multiparous (third to sixth delivery) females at 11 d post-partum with a weight of 4040 g.

We found a constant pattern in body weight throughout the study; a reduction after each delivery and a return to original weight before mating. For an animal like the rabbit with a relatively small fat reserve [19], it is crucial that weight does not drop below a critical level [36], in order to increase the survival probability of the female [37]. Taking into account our results, it seems that the critical body weight which increases the probability of survival should be around 4 kg.

It is well known that the development of rabbit females is related to the development of fetuses in the first reproductive cycles, i.e., for nulliparous and primiparous animals [38]. This agrees with the increment in PFT from 12 weeks of age until the second delivery. All females showed a PFT at first mating lower than 5.0. Martínez-Paredes et al. [39] described that a PFT at first mating above 6.0 mm should be avoided due to females being at higher risk of being culled. Moreover, PFT should be the most important trait that guarantees females’ reproductive longevity. 

It seems that body reserves are accumulated until a 7 mm PFT is reached no matter the reproductive status, which could be a threshold value to start managing these body reserves. Again, this would be an indicator of the need to maintain body reserves to increase the probability of survival [37]. In our experiment, this threshold is obtained from the third delivery. 

According to Theilgaard et al. [40], PFT can be a valuable indicator of how animals prioritise the distribution of their resources, and body condition is essential to maintaining rabbit females’ life functions while assuring the survival of their litters. Our results indicate that the correlation between weight and PFT is positive and low at any time. Although both traits are related to energy content, which is highly influenced by the size of the animal, all of them give useful information about the energy balance in the mid- to long-term [1].

An increase in NEFA concentration is interpreted as the short-time mobilization of energy [1]. NEFA concentrations are higher at delivery than at mating and on the 12th d of gestation, as expected due to higher energetic demand [9,37] and lower food ingestion of the female at that moment [23]. NEFAs are not correlated with weight, PFT, or leptin at any time. Similar results for weight and PFT were found in primiparous females at mating by Calle et al. [1] and low correlations were found at delivery. These results demonstrate the independence between the body reserves that females may have and their capacity to mobilise energy in times of energy deficit.

The highest levels of leptin are found at mating. Similar results have been shown in lines selected for daily gain in the fattening period [41]. Leptin can activate the secretion of hypothalamic luteinizing hormone–releasing hormone (LH-RH) [42,43], causing ovulation to be induced by coitus. The improvement of rabbit reproductive indices by leptin has suggested the potential use of this hormone at mating to improve fertility and prolificity [44]. We observed a decrease in leptin concentration between mating and delivery. During gestation, the lowest leptin values are obtained in the first third of gestation [45]. A substantial increase occurs from mid-gestation, followed by a decline after delivery [45]. A decrease in plasma’s leptin levels is usually associated with a reduced reproductive ability and the promotion of body reserve accretion at the expense of milk yield [41]. 

Our study estimated a positive and low correlation between body condition and leptin at mating and on the 12th d of gestation. These results at gestation agree with those reported in rabbits [45] and in humans [46]. Maffei et al. [47] showed that leptin may play an important role in regulating body weight by signaling the size of the adipose tissue mass.

Our experimental design is a common commercial farm practice when a semi-intensive rhythm is applied. Thus, the genetic material used was breeding stock in the rabbit breeding industry in the Mediterranean area [48] and females were fed with a standard commercial feed. Moreover, the number of kits was standardised as 9 for all lactation. Matching litters not only according to number but also according to the weight of kits during lactation is a practice widely used in commercial farms [49] and highly recommended to improve the survival of the rabbits [50]. Therefore, the results obtained in this trial could be extrapolated to the industry.

This study provides insight into how rabbits manage their body reserves and change their NEFA and leptin levels according to reproductive status. Based on this knowledge, it may be possible to develop new production strategies related to management, feeding, or reproduction.

## 5. Conclusions

Rabbit females which are able to maintain a semi-intensive reproductive rhythm during four parities weighted around 4 kg form first mating. Moreover, females increased perirenal fat thickness until the third delivery and maximised NEFA concentration at delivery and leptin concentration at mating.

Body weight, PFT, NEFA and leptin should be measured during critical moments of reproductive life in order to determine de body condition and energy mobilization, due to their low or null correlations.

## Figures and Tables

**Table 1:** Coefficients of correlation (±S.E.) between perirenal fat thickness (PFT), body weight, non-esterified fatty acid (NEFA) concentration, and leptin concentration at mating above diagonal and on the 12th d of gestation below diagonal.

	PFT	Body Weight	NEFA	Leptin
PFT		0.248 ± 0.020 *	0.119 ± 0.083	0.238 ± 0.031 *
Body weight	0.249 ± 0.035 *		−0.106 ± 0.056	0.342 ± 0.022 *
NEFA	−0.185 ± 0.097	−0.036 ± 0.041		0.056 ± 0.088
Leptin	0.105 ± 0.067	0.220 ± 0.047 *	0.015 ± 0.091	

* *p* < 0.05.

**Table 2:** Coefficients of correlation (±S.E.) between perirenal fat thickness (PFT), body weight, non-esterified fatty acids (NEFA) concentration, and leptin concentration at delivery.

	Body Weight	NEFA	Leptin
PFT	0.222 ± 0.020 *	−0.08 ± 0.087	−0.103 ± 0.061
Body weight		−0.09 ± 0.049	0.264 ± 0.054 *
NEFA			−0.106 ± 0.084

* *p* < 0.05.

## Data Availability

The data presented in this study are available on request from the corresponding author.
